# Natural Compounds with Antifungal Properties against *Candida albicans* and Identification of Hinokitiol as a Promising Antifungal Drug

**DOI:** 10.3390/antibiotics12111603

**Published:** 2023-11-08

**Authors:** Louis Camaioni, Bastien Ustyanowski, Mathys Buisine, Dylan Lambert, Boualem Sendid, Muriel Billamboz, Samir Jawhara

**Affiliations:** 1 CNRS, UMR 8576-UGSF-Unité de Glycobiologie Structurale et Fonctionnelle, INSERM U1285, F-59000 Lille, France; louis.camaioni@gmail.com (L.C.); bastien.ustyanowski@lacatholille.fr (B.U.); buisinemathys59320@gmail.com (M.B.); dylan.lambert2.etu@univ-lille.fr (D.L.); boualem.sendid@univ-lille.fr (B.S.); 2 Medicine Faculty, University of Lille, F-59000 Lille, France; 3 CHU Lille, Service de Parasitologie Mycologie, Pôle de Biologie Pathologie Génétique, F-59000 Lille, France; 4 INSERM, CHU Lille, Institut Pasteur Lille, U1167-RID-AGE-Facteurs de Risque et Déterminants Moléculaires des Maladies Liées au Vieillissement, University of Lille, F-59000 Lille, France; muriel.billamboz@junia.com; 5 JUNIA, Health and Environment, Laboratory of Sustainable Chemistry and Health, F-59000 Lille, France

**Keywords:** *Candida albicans*, hinokitiol, aromatic phenols, sesquiterpenols, cinnamic derivatives, *Caenorhabditis elegans*

## Abstract

*Candida albicans* is an opportunistic yeast that causes most fungal infections. *C. albicans* has become increasingly resistant to antifungal drugs over the past decade. Our study focused on the identification of pure natural compounds for the development of antifungal medicines. A total of 15 natural compounds from different chemical families (cinnamic derivatives, aromatic phenols, mono- and sesquiterpenols, and unclassified compounds) were screened in this study. Among these groups, hinokitiol (Hi), a natural monoterpenoid extracted from the wood of the cypress family, showed excellent anti-*C. albicans* activity, with a MIC value of 8.21 µg/mL. Hi was selected from this panel for further investigation to assess its antifungal and anti-inflammatory properties. Hi exhibited significant antifungal activity against clinically isolated fluconazole- or caspofungin-resistant *C. albicans* strains. It also reduced biofilm formation and hyphal growth. Treatment with Hi protected *Caenorhabditis elegans* against infection with *C. albicans* and enhanced the expression of antimicrobial genes in worms infected with *C. albicans*. Aside from its antifungal activities against *C. albicans*, Hi challenge attenuated the LPS-induced expression of pro-inflammatory cytokines (IL-6, IL-1β, and CCL-2) in macrophages. Overall, Hi is a natural compound with antifungal and anti-inflammatory properties, making Hi a promising platform with which to fight against fungal infections.

## 1. Introduction

The development of invasive fungal infections is a significant health problem in immunocompromised or hospitalized patients [1]. These fungal infections are associated with a high mortality and morbidity rate [2]. The most commonly identified *Candida* species in hospitals is *Candida albicans*. The rise in antifungal resistance around the world is one of the greatest emerging health problems, making it difficult to select effective antifungal treatments [3,4]. It has been established that the nematode *Caenorhabditis elegans* is one of the most successful in vivo approaches used to gain a better understanding of the pathogenesis of *Candida* infections and the innate immune response of the host [5,6]. Additionally, this nematode model is used to investigate the impact of antifungal compounds against *Candida* infection progression [6]. After the ingestion of yeast forms of *C. albicans* by the nematode, the yeast cells switch to a hyphal form in the liquid medium, resulting in tissue damage, serious infection, and even the death of the nematode [7].

Many strains of *C. albicans* are resistant to current antifungal drugs, including azoles and echinocandins [8]. A significant factor in the development of *C. albicans* resistance is extended exposure to antifungal drugs, particularly the excessive use of azoles and echinocandins as prophylaxis, or the empiric treatment of patients at risk of developing invasive candidiasis. *C. albicans* virulence is characterized by its ability to form biofilms, which are densely packed communities of cells that adhere to surfaces. In addition, the biofilms formed by *C. albicans* are highly resistant to a wide range of antifungal agents [6,8].

Nature, and particularly the plant kingdom, represent a great source of active ingredients. Essential oils are obtained from raw plant materials via physico-chemical processes such as dry distillation, or mechanical workout avoiding heating. The development of drugs from essential oils presents several challenges. Essential oils and natural extracts have traditionally been a rich source of therapeutic agents. However, researchers still have to overcome several hurdles, such as their complex chemical composition, limited availability, chemical stability, bioavailability, toxicity, or side effects. Natural extracts generally present complex chemical compositions, which depend on extraction methods (temperature, time, and solvent) and the plant itself (genus, species and part used).

It is therefore difficult to identify and characterize the active ingredients in such a mixture. In addition, natural compounds are often difficult to obtain in sufficient quantities for research and clinical trials due to their limited availability, owing to their low abundance in plants. The bioavailability, toxicity, as well as any potential side effects should also be thoroughly investigated. Additionally, from an ethical or environmental point of view, it should be validated that the natural compounds are not sourced from endangered or protected species. Therefore, we focused on identifying pure natural compounds as platform molecules for the development of next-generation antifungal drugs. Our selection process has already addressed these key points through the selection of compounds that are available in moderate to bulk quantities and are pure in form (purity greater than 95%), at a price which we have deemed reasonable for medicinal development (no more than EUR 1 per gram). This is why our work focused on the identification of pure natural compounds as platform molecules for the further development of next-generation antifungal drugs. It is noteworthy that each selected platform molecule contains a reactive group that is either a hydroxyl, carboxylic acid or a ketone group. 

Numerous bioresources and biological activities of essential oils have been reported, particularly regarding their antifungal activity [9]. The antimicrobial effects of thyme essential oil (*Thymus vulgaris*), containing thymol, have been reported [10,11,12]. The same statement applies to Oregano essential oil (*Origanum vulgare*), which contains active phenols and sesquiternes [13,14,15]. Peppermint essential oil (*Mentha piperita*), containing menthol and eucalyptol, is well known for its antifungal applications [16,17]. Lavender oil (*Lavandula angustifolia*), rich in linalool, geraniol, and eucalyptol, is also frequently used for its medicinal properties [18,19,20]. Other bioresources, such as clove, rosemary, tee-tree, eucalyptus, pine, cinnamon, and cumin, have also been studied. Among the various extracts derived from cypress wood, hinokitiol (β-thujaplicin), a natural extract from the Japan cypress, is a monoterpene compound. Hinokitiol has been shown to exert a wide range of biological and pharmacological effects, including antimicrobial and ant-inflammatory properties [21,22,23], neuroprotective activities [24], and anticancer effects [25].

In the present study, the compound with the best MIC from the first screening of 15 selected compounds was assessed for its antifungal activity against *Candida* viability, *Candida* biofilms, and antifungal-resistant clinical *Candida* isolates, as well as in a model of *C. elegans* infection. In addition to its antifungal activity against *C. albicans*, the anti-inflammatory property of this compound was also assessed in macrophages.

## 2. Results

In the present study, 15 pure natural compounds derived from essential oils were selected and evaluated for their potential antifungal effects against *C. albicans*. A variety of criteria were used to select these molecules, including their physicochemical properties, their ability to serve as platform molecules for chemical modifications, e.g., the presence of hydroxyl, carboxylic acid or ketone groups, their availability in fair to bulk quantities, their price, and their ADMETs (adsorption, distribution, metabolism, excretion and toxicity). Table 1 provides a summary of the chemical structures and physicochemical descriptors for each compound. SwissADME software was used for the prediction of the physicochemical descriptors (ADME properties are described in detail in Section 4). 

Thus, based on literature reports and in silico calculations, 15 compounds belonging to four classes of molecules were selected, and their potential for inhibiting *C. albicans* growth was evaluated through a series of tests. The MIC values of each compound are shown in Table 1.

The first sub-class is composed of derivatives of cinnamic acid, which occur naturally in plants. In the selection process, ferulic, caffeic, and syringic acids, bearing an aromatic ring as well as an acrylic acid, were chosen. The MIC values for these three molecules were around 1000 µg/mL, which means that they displayed a low inhibitory capacity. However, from a chemical point of view, there was an increase in the biological activity of compounds with hydroxy groups attached to the aromatic moiety. There is no doubt that caffeine (2 OH) is a more effective antioxidant than ferulic acid (1 OH + 1 OCH_3_), which in turn is more effective than sinapic acid (1 OH and 2 OCH_3_). The activity of the compounds decreased as their lipophilicity increased. It was evident that when sinapic acid was compared to syringic acids, there was a decrease in activity as a result of the double bond (acrylate). Altogether, the derivatives of cinnamic acid did not seem to be interesting candidates for use as antifungal agents against *C. albicans*.

The second family is composed of aromatic phenols, such as eugenol, thymol, and sesamol. Overall, the MICs for aromatic phenols were better than those for cinnamic acid derivatives, ranging from 660 to 820 µg/mL. Eugenol and ferulic acid are both composed of the same 3-methoxy, 4-hydroxyphenyle aromatic moiety. In view of this similarity, it can be concluded that the carboxylic acid in ferulic acid has a deleterious effect on its biological activity. In this family, sesamol had the highest hydrophilicity and solubility, as well as the best MIC value of 660 µg/mL. There was a significant correlation between the lowest log P and the most active molecule in both series.

The third family contains four mono- and one sesquiterpenols (e.g., L-menthol, citronellol, geraniol, linalool, and cedrol, respectively). These are hydroxylated linear or cyclic alkyles. The monoterpenols exhibited the same low MIC value of 780 µg/mL, which proves that the position of the alcohol moiety and the stereochemistry are not of crucial significance to the activity of the monoterpenol. Cedrol, which is a hindered cyclic alkyl alcohol, exhibited a moderate MIC of 111 µg/mL, which is better than the MICs of all monoterpenols that were tested. Altogether, members of this third family are of moderate interest as antifungal agents, but cedrol could potentially be considered as a platform molecule for further development or optimization.

The fourth family of compounds consists of three chemical compounds, namely kojic acid, which is a hydroxy-pyrone molecule, hinokitiol (Hi), a tropolone derivative, and eucalyptol, also known as 1,8-cineole, which is a bicyclic ether that belongs to the monoterpenoids. Although kojic acid possessed excellent hydrophilicity and solubility, it did not appear to have any antifungal activity against *C. albicans*. There was no evidence that eucalyptol exhibited any potent antifungal activity. However, Hi, a moderately lipophilic aromatic seven-membered tropolone isolated from the *Cupressaceae* family, displayed excellent anti-*Candida* activity when tested, with a MIC of 8.21 µg/mL in our screening. In this regard, Hi was selected from this panel to conduct further studies.

### 2.1. Analysis of the Antifungal Properties of Hi against Clinical Strains of C. albicans

**Figure 1:**
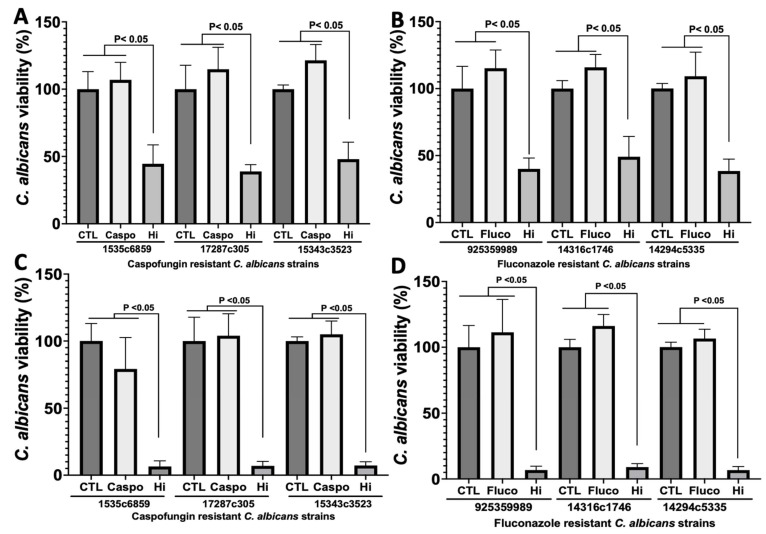
Influence of Hi on the viability of *C. albicans* isolates resistant to caspofungin and fluconazole. (**A**,**C**) Caspofungin-resistant *C. albicans* strains. (**B**,**D**) Fluconazole-resistant *C. albicans* strains. Clinical isolates of *C. albicans* were challenged with Hi at 1× MIC (**A**,**B**) and 2× MIC (**C**,**D**). The viability of *C. albicans* was evaluated after 24 h using Alamar Blue reagent. CTL: Clinical strain of *C. albicans* without antifungal treatment; Fluco: *C. albicans* cells challenged with fluconazole; Caspo: *C. albicans* cells challenged with caspofungin.

The effectiveness of Hi against fluconazole- and caspofungin-resistant *C. albicans* strains isolated from patients was assessed in order to determine whether Hi can eradicate these drug-resistant clinical isolates (Table 2). Fluconazole- and caspofungin-resistant *C. albicans* strains displayed a significant reduction in viability with Hi challenge (Figure 1). Thus, according to these data, it appears that Hi can inhibit drug-resistant *C. albicans* strains, which is one of the major concerns when treating patients with invasive *Candida* infection.

### 2.2. Effect of Hi on C. albicans Biofilm and Hyphal Formation

**Figure 2:**
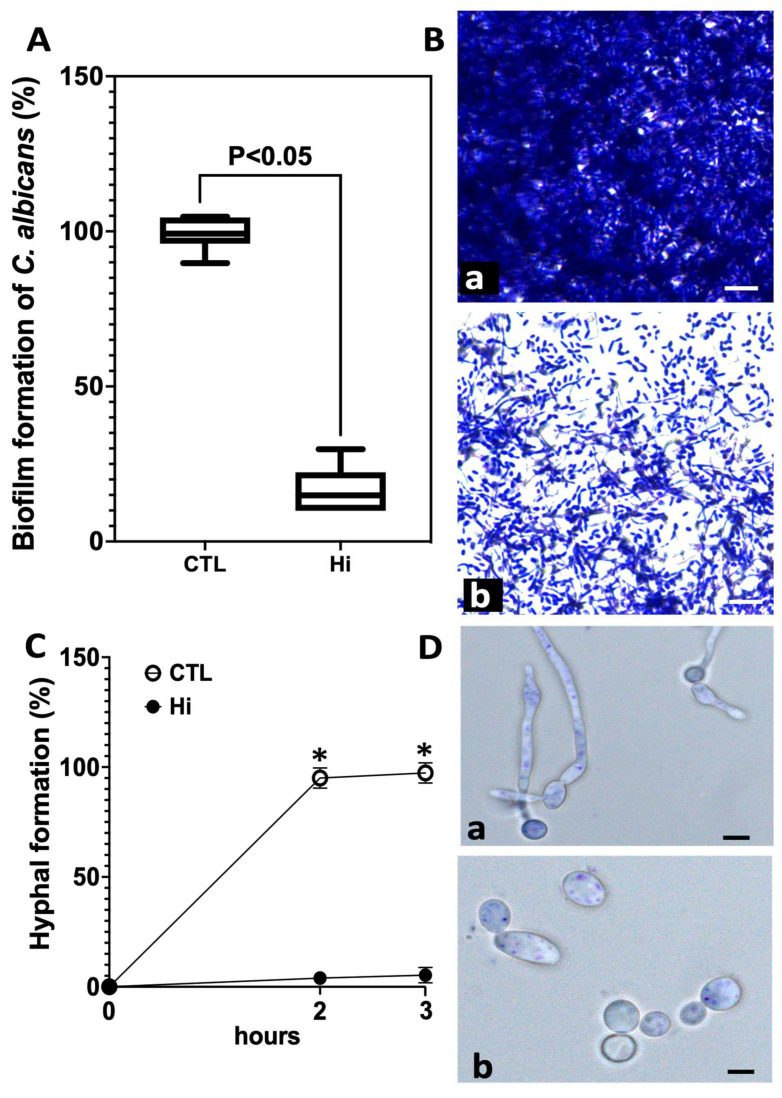
Impact of Hi challenge on *C. albicans* biofilm formation and hyphal growth. (**A**) The cells of *C. albicans* developed into a dense biofilm after 48 h. Hi at 1× MIC was added to the *C. albicans* biofilms for 24 h. CTL: *C. albicans* alone without antifungal challenge; (**B**) biofilms of *C. albicans* challenged with (a) CTL: *C. albicans* alone without antifungal treatment, (b) Hi. Scale bars represent 100 µm. (**C**) Hyphal formation of *C. albicans*. *C. albicans* cells were monitored at 0, 2, and 3 h after hyphal formation in RPMI-1640 medium containing 10% FBS. (**D**) Microscopic observation of hyphal formation after 3 h incubation. (a) *C. albicans* cells without treatment. (b) *C. albicans* challenged with Hi. * *p* < 0.05 for control conditions vs. *C. albicans* challenged with Hi. Scale bars represent 5 µm.

To determine whether Hi has the potential to affect *C. albicans* biofilm formation, which is implicated in various antifungal resistance mechanisms, including resistance to azoles and echinocandins, *C. albicans* biofilms were challenged with Hi at 1× MIC (Figure 2). Hi inhibited the formation of *C. albicans* biofilms by 90%. Microscopic examination revealed that the biofilm matrix of *C. albicans* was dense and highly compacted when it was challenged with phosphate-buffered saline (PBS) as a control (CTL). In contrast, when the *C. albicans* cells were incubated with Hi, the biofilm seemed to dissolve, and the *C. albicans* cells appeared to separate from the biofilm matrix.

The transition from yeast to hyphae is one of the virulence factors associated with the pathogenicity of *C. albicans* (Figure 2). It was therefore assessed whether Hi has an impact on the hyphal formation of *C. albicans*. Hyphal formation was induced in RPMI-1640 medium containing 10% fetal bovine serum (FBS), and *C. albicans* cells were monitored at 0, 2, and 3 h. After 2 h of incubation at 37 °C, vigorous hyphal formation was observed in the untreated cells. In contrast, Hi challenge reduced hyphal formation at 2 h. Its effect was still clearly effective at preventing hyphal formation even after 3 h of exposure (Figure 2).

### 2.3. Effect of Hi Treatment on the Survival of C. elegans Infected with C. albicans

**Figure 3:**
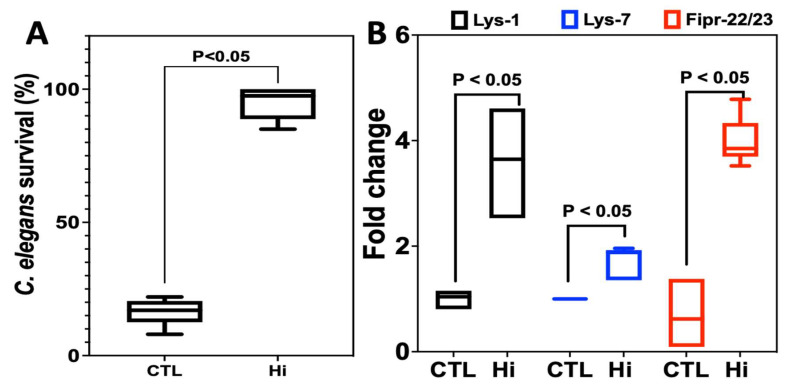
Impact of Hi on *C. albicans* pathogenesis in a *C. elegans* infection model. (**A**) The survival rate of nematodes infected with *C. albicans* was evaluated every day for four days, and the percentage of worms that survived on day four was determined. Nematodes infected with *C. albicans* were treated with Hi at 1× MIC (8.21 µg/mL). Nematodes that failed to respond to contact were determined to be dead using a platinum wire pick. CTL: *C. albicans*-infected nematodes without antifungal treatment; Hi: *C. albicans*-infected nematodes treated with Hi. (**B**) Antimicrobial peptide expression in *C. elegans* infected with *C. albicans* (Lys-1, Lys-7, and Fipr-22/23). The adult nematodes were infected for 6 h with *C. albicans* SC5314 lawns and then treated for 12 h with Hi at 1× MIC (8.21 µg/mL). CTL represents *C. albicans*-infected nematodes without treatment. Hi corresponds to *C. albicans*-infected nematodes treated with Hi. Values are shown as mean ± SD of four independent experiments.

Using a model of *C. elegans* infected with *C. albicans*, we were able to determine whether Hi was effective in vivo against *C. albicans* (Figure 3). Adult nematodes infected with *C. albicans* were treated with Hi at a concentration of 1× MIC. The survival rate of *C. elegans* was monitored daily via microscopic observation. During the experiment, nematodes that were infected with *C. albicans* without treatment were used as a CTL group. An 85% nematode mortality rate within 4 days of fungal infection was recorded. When the worms were treated with Hi, there was a significant increase in the survival rate of *C. elegans* compared to the untreated CTL group of worms. A 95% survival rate was observed for nematodes treated with Hi. To investigate the effect of Hi on the immune response of *C. elegans*, the expression of Lys-1 and Lys-7, as well as Fipr-22/23, which are involved in the antimicrobial response, was examined. The Hi treatment of *C. elegans* infected with *C. albicans* was shown to enhance the expression of the antimicrobial genes Lys-1, Lys-7, and Fipr-22/23 when compared to untreated nematodes (CTL). This indicates that Hi treatment improved the expression of antimicrobial genes promoting the elimination of *C. albicans* (Figure 3).

### 2.4. Anti-Inflammatory Properties of Hi

**Figure 4:**
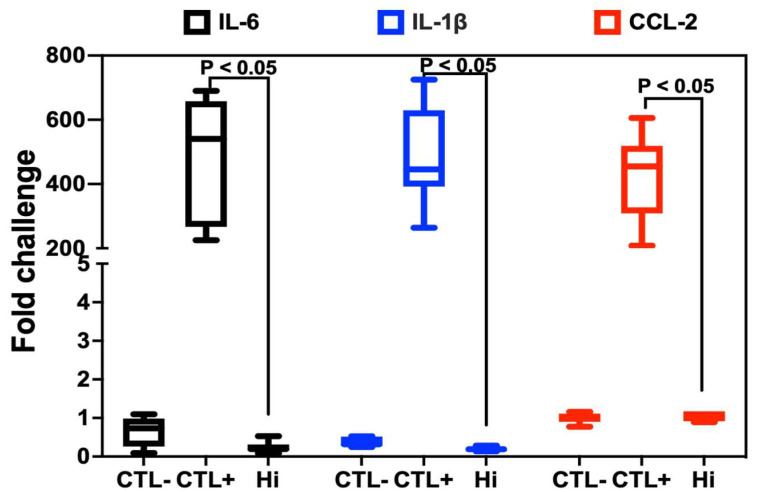
Expression of proinflammatory mediators in macrophages challenged with lipopolysaccharide (LPS) and treated with Hi. Relative expression levels of IL-6, IL-1β, and CCL-2 mRNA, respectively, in macrophages. CTL−: control group (macrophages alone); CTL+: macrophages exposed to LPS; Hi: macrophages challenged with LPS and treated with Hi.

An excessive pro-inflammatory response can lead to chronic inflammation and disrupt the pathways responsible for maintaining biological homeostasis, resulting in a variety of detrimental health problems. We assessed whether Hi modulated LPS-induced proinflammatory responses in macrophages (Figure 4). A significant reduction in the expression of cytokines such as IL-1β, IL-6, and chemokine CCL-2 was found after the challenge of macrophages with Hi.

## 3. Discussion

A panel of natural molecules derived from essential oils was selected based on their physicochemical properties and each individual compound was tested for its potential antifungal activity against *C. albicans*. Through a series of analyses, 15 compounds from four classes (cinnamic derivatives, aromatic phenols, mono- and sesqui-terpenols, and other unclassified molecules) were evaluated. Considering the MIC values of cinnamic acid derivatives, it does not appear that these compounds are of particular interest as antifungal candidates against *C. albicans*. While the MIC values for aromatic phenol derivatives and mono and sesquiterpenoids were better than those obtained for the cinnamic acid derivatives, these two families of compounds are only of moderate interest as antifungal agents against *C. albicans*. Among the fourth group of unclassified compounds, Hi showed excellent anti-*Candida* activity, with a MIC value of 8.21 µg/mL. In light of these data, Hi was selected from this panel for further investigation to assess its antifungal properties against *C. albicans*. Hi is a natural monoterpenoid derived from wood of the cypress family [54]. A tropolone core containing an isopropyl group forms the backbone of its chemical skeleton. Hi is found abundantly in *Thujopsis dolabrata*, which is a dense and evergreen conifer native to the Central Japan region. It is known to have biological activity, including antitumor properties [55,56,57]. Several studies have shown that Hi reduces tumor growth by activating apoptosis and autophagy [25,56,58]. Hi has been shown to possess significant antibacterial activity against a range of pathogenic bacteria [23,59]. In addition to its antibacterial activities, Hi has also been assessed against a variety of fungal species, including *Aspergillus* and *Candida* species [60,61].

In the present study, a significant reduction in *C. albicans* viability was observed after the Hi challenge of fluconazole and caspofungin-resistant strains, indicating that this compound has potential as a natural antifungal agent in the case of antifungal drug resistance. These data are in accordance with a previous study, which showed that Hi had antifungal activity against fluconazole-resistant *Candida* strains [61]. Throughout this study, Hi demonstrated significant anti-*Candida* activity against all fluconazole-resistant strains that were tested [61]. In terms of biofilm formation, Hi was shown to significantly inhibit *C. albicans* biofilm formation by 90%. Furthermore, it was found that *C. albicans* cells were dispersed from the biofilm matrix after being challenged with Hi, indicating that the biofilm was dissolving and the *C. albicans* cells were being separated from the matrix. In line with this study, Kim et al. showed that Hi efficiently prevented biofilm formation in both fluconazole-susceptible and fluconazole-resistant *Candida* strains [61]. In addition to biofilm formation, one of the virulence factors of *C. albicans* is the formation of hyphae. Hi challenge was found to reduce hyphal formation in *C. albicans*. It has been shown that Hi decreases the expression levels of UME6 and HGC1 in *C. albicans*, which are responsible for long-term hyphal growth [61]. Furthermore, Hi has been shown to suppress the expression of the gene *CYR1*, which encodes the component of the signaling pathway that controls hyphal formation, cAMP-PKA, as well as the expression level of CYR1 [61]. A model of *C. elegans* infected with *C. albicans* showed that worms treated with Hi had a higher survival rate when compared to a CTL group of untreated worms. *C. elegans* survival is consistent with the observations regarding hyphal growth, which showed that Hi challenge inhibited *C. albicans* filamentation, which in turn prevented fungal infection. The treatment of *C. elegans* with Hi after *C. albicans* infection significantly enhanced the expression of the antimicrobial genes Lys-1, Lys-7, and Fipr-22/23 when compared to untreated nematodes. This indicates that Hi treatment increases the expression of antimicrobial genes, thereby facilitating the elimination of the parasite.

In addition to its antifungal properties against *C. albicans*, we also determined whether Hi could modulate the LPS-induced expression of proinflammatory genes in macrophages. The expression of cytokines such as IL-1β, IL-6, and chemokine CCL-2 was significantly reduced after the challenge of macrophages with Hi. Different studies corroborate our findings that Hi challenge suppresses the expression of pro-inflammatory cytokines in macrophages [21,62].

In conclusion, Hi showed excellent antifungal activity against *C. albicans*, including clinical isolates of *C. albicans* that were resistant to the most common antifungal agents. Furthermore, Hi also reduced biofilm formation and hyphal growth. Treatment with Hi protected *C. elegans* against infection with *C. albicans* and enhanced the expression of antimicrobial genes. In addition to its antifungal properties against *C. albicans*, Hi challenge attenuated the LPS-induced expression of pro-inflammatory cytokines in macrophages, indicating its anti-inflammatory properties. Overall, Hi is a natural compound that has antifungal and anti-inflammatory properties, making it a promising candidate for the development of new drugs able to treat fungal infections and to prevent the spread of antifungal resistance to synthetic compounds.

## 4. Materials and Methods

### 4.1. Fungal and Human Cell Line Culture

The strain used for this study was *C. albicans* SC5314 (Table 1) [52]. *C. albicans* yeast cells were cultured on Sabouraud dextrose agar (SDA) at 37 °C for 24 h [63]. A suspension of *C. albicans* was prepared by culturing the yeast cells in Sabouraud dextrose broth (Sigma-Aldrich, Saint-Quentin Fallavier, France) at 37 °C for 24 h in a rotating shaker. A series of washes with PBS (phosphate-buffered saline) were performed on the yeast cells before being centrifuged at 2500 rpm for 5 min and resuspended in PBS. Regarding the clinical strains of *C. albicans*, six strains isolated from patients with a history of fluconazole or caspofungin resistance were cultured on SDA for 24–48 h. The MIC values were evaluated based on the laboratory standard culture microdilution method developed by the Clinical and Laboratory Standard Institute (CLSI), as described previously by Pfaller et al. (Table 2) [64]. In terms of identifying the clinical isolates, a volume of 1.5 µL of matrix solution (α-cyano-4-hydroxycinnamic acid; Bruker Daltonics, Bremen, Germany) with 50% acetonitrile, 47.5% water, and 2.5% trifluoroacetic acid was added to each colony of *C. albicans* clinical isolate. Each strain of *C. albicans* was identified using MALDI-TOF MS (Microflex-Bruker Daltonics). For the culture of macrophages, the differentiation of THP-1 cells (human leukemia monocytic cell line) into macrophages was achieved using phorbol-12-myristate13-acetate (PMA: Sigma-Aldrich) at a concentration of 200 ng/mL for 72 h [65,66]. Macrophages at a concentration of 10^6^ cells/well were then incubated for 24 h in RPMI medium. Afterwards, macrophages were exposed to lipopolysaccharide (LPS) at a concentration of 250 ng/mL (LPS from *Escherichia coli* O111:B4; Sigma-Aldrich) for 6 h [67]. In addition, Hi at a concentration of 1× MIC (8.21 µg/mL) was added to macrophages exposed to LPS. Next, macrophages were harvested and resuspended in RA1 buffer for mRNA extraction, followed by RT-PCR and q-PCR [63,68]. SYBR green real-time PCR master mix reagent was used to amplify cDNAs for quantitative PCR. A one-step software program was used to determine the SYBR green dye intensity.

### 4.2. Fungal Viability Assays

JUNIA supplied all natural compounds from (TCI, Paris, France) and (Sigma-Aldrich, Saint-Quentin Fallavier, France). For the selection of the compounds, SwissADME software was used for the prediction of the physicochemical descriptors [14]. These descriptors are meaningful criteria used to select candidates to be evaluated further. Log P describes how the molecules split themselves between a water/oil biphasic medium. Therefore, log P helps to predict how the drug candidates will behave in the body. The degree of lipophilicity of a compound is a very significant determinant of its absorption, distribution in the body, penetration through membranes, metabolism, and elimination (ADME properties) [15,16]. Log*Kp* represents the skin permeation factor. This is of great interest to develop compounds that could be used as topical formulations as it gives an indication of the absorption rate. When log*Kp* is low, diffusion through the skin is slow. Citronellol was the compound exhibiting the fastest rate of permeation, with a *Kp* of 3.3 × 10^−5^ cm s^−1^; this is compared to kojic acid, which displayed a *Kp* of 3.4 × 10^−8^ cm s^−1^, indicating that kojic acid diffuses 1380 times slower than citronellol among our panel of selected compounds.

The topological polar surface area (TPSA) can be used in drug development to estimate the effectiveness of a drug in terms of its ability to penetrate cells. A TPSA > 140 angstroms squared (Å^2^) is generally considered to be a poor permeation barrier for molecules. Our panel of compounds followed this metric. Furthermore, molecules with a low TPSA can permeate the blood–brain barrier (BBB).

It is of critical importance for ADME properties to have a high level of solubility in water. Among our panel of selected compounds, it is noteworthy that some of them were highly soluble (e.g., kojic acid and sesamol), while many of them have limited solubility, thus limiting their effectiveness (e.g., cedrol, geraniol, and citronellol).

Gastrointestinal absorption (GIA) refers to the absorption of compounds through the gut, whereas BBBP refers to their permeation through the blood–brain barrier. These data will help us to understand the possible distribution of molecules in the body. In addition, it is noteworthy that all compounds have a high gastrointestinal absorption rate but a variable BBBP level.

Each compound was divided into multiple small batches and stored at −20 °C. A fresh aliquot for each experiment was diluted in PBS and adjusted to the appropriate concentration. MIC assays for these natural compounds were carried out using Alamar Blue reagent (Thermo Fisher Scientific, Waltham, MA, USA) [69]. The MICs of caspofungin and fluconazole were also used as positive controls for these experiments. Since Alamar Blue is metabolized by the yeast, it can be used to determine both the MIC value and the activity of the fungal cells. Alamar Blue (10 µL) was placed in each well containing 5 × 10^3^ yeasts in 90 µL of RPMI medium. As for the antifungals, different concentrations of natural compounds were used (between 5 × 10^−3^ M and 5 × 10^−6^ M in the well). The MIC was determined for each natural compound as the concentration that inhibited yeast growth by 99%. A Fluostar OMEGA spectrophotometer was used to measure the absorbance of each sample at 600 nm at both T0 and T24. Additionally, a volume of 100 µL of each dilution was grown on SDA and incubated for 48 h to assess the fungal viability.

### 4.3. C. albicans Biofilm Formation

In this stage, 5 × 10^3^ *C. albicans* yeast cells were suspended in 200 µL of RPMI medium with 10% fetal bovine serum (FBS). This suspension was then placed in each well of a polystyrene plate (Greiner Bio-One). Following the incubation of the plates for 48 h at 37 °C, the plates were washed several times with PBS to eliminate yeast cells that were not adherent to the plates [70]. Hi was then added to the plates at a concentration of 1× MIC (8.21 µg/mL) for the next 24 h. After 24 h of incubation, the wells were washed with PBS and air dried at 37 °C. The biofilms were stained with 0.4% crystal violet solution (Fluka) for 20 min. The wells were then washed multiple times with PBS, and a volume of 200 µL of ethanol was added to each well. The absorbance of the destaining solution, which represents the number of viable *C. albicans* cells, was determined at 550 nm using a spectrophotometer (FLUOstar; BMG Labtech). The average of six replicates of two independent experiments represents the *C. albicans* biofilm data. For the induction of hyphal growth, a suspension of 5 × 10^3^ *C. albicans* yeast cells was challenged with Hi at a concentration of 1× MIC (8.21 µg/mL) in 200 µL of RPMI medium with 10% FBS and incubated at 37 °C for 3 h. *C. albicans* cells were monitored at 0, 2, and 3 h. Germ tube or hyphal formation was assessed microscopically using a Zeiss AxioImager microscope [53].

### 4.4. C. elegans Survival Assay

The culture of *C. albicans* SC5314 in Sabouraud dextrose broth was maintained for 24 h. A lawn of *C. albicans* was cultured by spreading a volume of 10 µL of *C. albicans* yeast cell suspension on brain heart infusion plates containing amikacin (45 µg/mL). The plates were then placed at 37 °C for 24 h. A wild-type strain *C. elegans* N2 was cultured in nematode growth medium seeded with *E. coli* strain OP50 at 20 °C. The populations of *C. elegans* were synchronized and kept at 20 °C for incubation [71]. For each experiment, 100 nematodes were selected. To eliminate *E. coli* from the worms, the nematodes were washed with M9 buffer containing amikacin at a concentration of 90 µg/mL before being transferred to *C. albicans* lawns. After the incubation of plates at room temperature for 6 h, multiple washes with M9 buffer were then performed on the worms to ensure that all *C. albicans* yeast cells were removed from their cuticles. A total of 70–80 worms infected with *C. albicans* were then placed in wells in a 6-well microtiter dish containing 20% brain heart infusion, 2 mL of 80% liquid M9 buffer, 90 µg/mL of amikacin, and 10 µg/mL of cholesterol in ethanol. Hi at 1× the MIC value (8.21 µg/mL) was then added to each well. The incubation of the plates was carried out at room temperature for 12 h. During the course of the experiment, the worms were examined daily for survival for a period of 4 days, and if no nematodes responded to mechanical stimulation with a pick, they were considered dead. In terms of the RT-PCR assay, the total RNA was extracted from nematodes after 12 h of Hi challenge using a NucleoSpin RNA^®^ kit (Macherey-Nagel, Hoerdt, France). RNA from worms was measured via spectrophotometry (Nanodrop; Nyxor Biotech, Paris, France). To synthesize the cDNA, a high-capacity DNA reverse transcription (RT) kit (Applied Biosystems, Foster City, CA, USA) was employed, along with the Master Mix (Applied Biosystems). The cDNA was amplified using Fast SYBR green (Applied Biosystems) in a one-step method (Applied Biosystems). An analysis of the SYBR green dye intensity was performed using software that was designed for one-step analysis. The results were normalized to the reference gene, *act-2*.

### 4.5. Statistical Analysis

To determine the differences between the groups, the Mann–Whitney U test was used. The data were considered statistically significant when the *p* value was *p* < 0.05; *p* < 0.01; and *p* < 0.001. All statistical analyses were performed using version 10 software of GraphPad Prism (GraphPad, La Jolla, CA, USA).

## Figures and Tables

**Table 1:** Panel of natural compounds, their MICs against *C. albicans* and physicochemical descriptors.

	N°	Compound	Structure	M (g/mol)	MIC (µg/mL)	log*P* ^a^	log*Kp* cm/s ^b^	TPSA (Å^2^) ^c^	BBBP ^d^	Ref ^e^
CINNAMIC DERIVATIVES	1	Ferulic acid	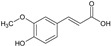	194.18	970.9	1.36	−6.41	66.76	Yes	[26,27]
	2	Caffeic acid	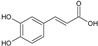	180.16	900.8	0.93	−6.58	77.76	No	[28,29]
	3	Sinapic acid	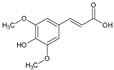	224.21	1121.05	1.31	−6.63	75.99	No	[30,31]
AROMATIC PHENOLS	4	Syringic acid	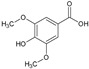	198.17	990.85	0.99	−6.77	75.99	No	[20]
	5	Eugenol	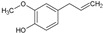	164.20	821	2.25	−5.69	29.46	Yes	[32]
	6	Sesamol	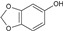	132.11	660.55	1.19	−6.27	38.69	Yes	[33,34]
	7	Thymol	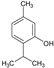	150.22	751.1	2.80	−4.87	20.23	Yes	[35,36]
MONO & SESQUITERPENOLS	8	L-Menthol	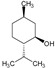	156.27	781.35	2.59	−4.84	20.23	Yes	[37,38]
	9	Citronellol	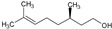	156.27	781.35	2.92	−4.48	20.23	Yes	[38,39]
	10	Geraniol	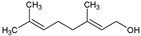	154.25	781.35	2.74	−4.71	20.23	Yes	[40,41]
	11	Linalool	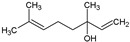	154.25	781.35	2.66	−5.13	20.23	Yes	[42,43]
	12	Cedrol	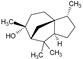	222.37	111.185	3.54	−4.90	20.23	Yes	[44,45]
OTHERS	13	Kojic acid	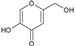	142.11	710.55	−0.16	−7.62	70.67	No	[46,47,48]
	14	Hinokitiol	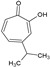	164.20	8.21	1.98	−5.79	37.30	Yes	[49]
	15	Eucalyptol	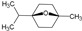	154.25	781.35	2.77	−5.13	9.23	Yes	[50,51]

^a^ Log P: octanol/water partition coefficient calculated using SwissADME; ^b^ Log*Kp*: skin permeation calculated using SwissADME; ^c^ TPSA: topology polar surface area calculated using SwissADME; ^d^ BBBP: blood–brain barrier permeant; ^e^ Ref: references.

**Table 2:** MIC values of the *C. albicans* strains used in the study.

Strain	Description	Caspofungin MIC (µg/mL)	Fluconazole MIC (µg/mL)	Ref.
*C. albicans* SC5314	Wild-type	0.03	0.5	[52]
*C. albicans* 15343c3523	Blood, caspofungin-resistant	2	0.5	[53]
*C. albicans* 15351c6859	Venous catheter, caspofungin-resistant	4	1	[53]
*C. albicans* 92535989	Tracheal secretion, fluconazole-resistant	0.06	64	[53]
*C. albicans* 17287c305	Blood, caspofungin-resistant	8	0.5	[53]
*C. albicans* 14316c1746	Bronchoalveolar lavage, fluconazole-resistant	0.03	128	[53]
*C. albicans* 14294c5335	Stools, fluconazole-resistant	0.06	5	[53]

## Data Availability

The data presented in this study are available in the article.
